# Shared genetic architecture and causal relationship between liver and heart disease

**DOI:** 10.1016/j.isci.2024.109431

**Published:** 2024-03-06

**Authors:** Ziyi Fang, Sixiang Jia, Xuanting Mou, Zhe Li, Tianli Hu, Yiting Tu, Jianqiang Zhao, Tianlong Zhang, Wenting Lin, Yile Lu, Chao Feng, Shudong Xia

**Affiliations:** 1 Department of Gastroenterology, The Fourth Affiliated Hospital of School of Medicine, and International School of Medicine, International Institutes of Medicine, Zhejiang University, Yiwu 322000, China; 2 Department of Cardiology, The Fourth Affiliated Hospital of School of Medicine, and International School of Medicine, International Institutes of Medicine, Zhejiang University, Yiwu 322000, China; 3 Department of Orthopedics, The Second Affiliated Hospital of Wenzhou Medical University, Wenzhou, China; 4 Department of Critical Care Medicine, The Fourth Affiliated Hospital of School of Medicine, and International School of Medicine, International Institutes of Medicine, Zhejiang University, Yiwu 322000, China

**Keywords:** Non-infectious disease, Genetics, Bioinformatics

## Abstract

This study investigates the relationship and genetic mechanisms of liver and heart diseases, focusing on the liver-heart axis (LHA) as a fundamental biological basis. Through genome-wide association study analysis, we explore shared genes and pathways related to LHA. Shared genetic factors are found in 8 out of 20 pairs, indicating genetic correlations. The analysis reveals 53 loci with pleiotropic effects, including 8 loci exhibiting shared causality across multiple traits. Based on SNP-p level tissue-specific multi-marker analysis of genomic annotation (MAGMA) analysis demonstrates significant enrichment of pleiotropy in liver and heart diseases within different cardiovascular tissues and female reproductive appendages. Gene-specific MAGMA analysis identifies 343 pleiotropic genes associated with various traits; these genes show tissue-specific enrichment primarily in the liver, cardiovascular system, and other tissues. Shared risk loci between immune cells and both liver and cardiovascular diseases are also discovered. Mendelian randomization analyses provide support for causal relationships among the investigated trait pairs.

## Highlights

• The discovery establishes a clear genetic correlation between certain liver and heart diseases
• The findings offer new insights into the biological mechanisms behind liver and heart diseases
• The study provides evidence for pleiotropy in certain genetic loci affecting both liver and heart health

## Graphical abstract

**Figure:**
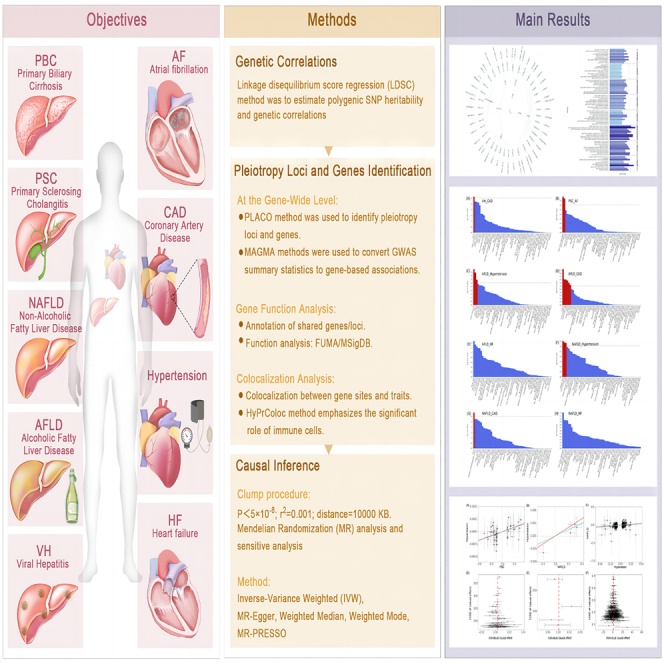

## Introduction

Organ interactions play a crucial role in maintaining internal homeostasis and facilitating adaptive responses to disease.^1^^,^^2^ Among these interactions, the liver and heart exhibit a complex bidirectional relationship known as the liver-heart axis (LHA), which has garnered significant research interest.^3^^,^^4^^,^^5^

Scholars, both domestically and internationally, have devoted significant efforts to investigate the therapeutic targets of LHA. Several studies have provided evidence that nonalcoholic fatty liver disease (NAFLD) accelerates the progression of coronary atherosclerosis, leading to the development and advancement of coronary artery disease (CAD).^6^^,^^7^ However, our current understanding of LHA remains incomplete. Existing literature suggests that the underlying pathogenic mechanisms of LHA may arise from systemic inflammatory responses, dysbiosis in gut microflora, endothelial dysfunction (ED), oxidative stress, and other factors,^4^^,^^8^^,^^9^^,^^10^ most of which have a genetic predisposition.^4^ Inspired by the concept of shared genetic etiology, it is plausible that there are associations between liver and heart diseases.

Genome-wide association studies (GWASs) have successfully identified a wide range of genetic variants, providing a comprehensive understanding of the genes involved in the development, progression, and treatment of diseases.^11^^,^^12^^,^^13^ Gong et al. utilized the GWAS database to uncover the shared genetic influence of the gut-brain axis (GBA) and conducted an analysis of pleiotropy.^14^ Their research has opened up avenues for investigating common genetic loci associated with LHA and established a concurrent causal relationship between these two conditions. These findings will offer new insights and perspectives for clinicians to intervene or simultaneously address both disorders in clinical practice.

In this genome-wide pleiotropic association study, we conducted a comprehensive analysis using large-scale GWAS aggregated data to investigate the potential genetic associations between five liver diseases (primary biliary cirrhosis [PBC], primary sclerosing cholangitis [PSC], NAFLD, alcoholic fatty liver disease [AFLD], viral hepatitis [VH]) and four heart diseases (heart failure [HF], CAD, Hypertension, atrial fibrillation [AF]). We will utilize appropriate tools to initially evaluate the genetic links between these two disease groups.^15^ Futher, to improve comprehension of the shared genetic structure of disease pairs in the LHA, the genome-wide SNVs and gene levels were systematically examined using various genetic statistical methods to explore multiple associations of potency, while also investigating sequential biological pathways. The shared genetic etiology often exhibits pleiotropy; therefore, in order to identify pleiotropic loci, we conducted additional analyses including gene set enrichment analysis, pathway analysis, tissue-specific analysis, cell-specific analysis, and investigation of immune cells that may be involved in the co-localized associations.^16^^,^^17^^,^^18^^,^^19^ Mendelian randomization (MR) analyses were subsequently performed to assess the causal relationship between liver and heart disease.^20^^,^^21^

## Results

### Genetic correlation between liver and heart disease

The results obtained through linkage disequilibrium score regression (LDSC) are presented in Table 1(Figure S1). We conducted characterization and analysis to identify eight pairs of traits related to liver and heart disease that exhibited significant genetic correlations. These pairs were AFLD-CAD (p = 0.002), AFLD-HF (p = 8.00E-04), AFLD-Hypertension (p = 0.027), NAFLD-CAD (p = 2.00E-04), NAFLD-HF (p = 0.006), NAFLD-Hypertension (p = 0.008), PSC-AF (p = 0.007), and VH-CAD (p = 0.014). The stability of most results was confirmed through high-definition likelihood (HDL) analysis. Additionally, Bonferroni correction was applied for multiple testing (p < 0.05/20 = 0.0025). Three pairs (AFLD-CAD, AFLD-HF, and NAFLD-CAD) still showed significant genetic correlations.

**Table 1:** Genetic correlation analysis results

Trait pair	LDSC			HDL	
	rg (SE)	p	Intercept (SE)	rg (SE)	p
AFLD-AF	−0.019 (0.058)	0.737	0.003 (0.005)	−0.031 (0.073)	0.666
AFLD-CAD	0.183 (0.06)	**0.002**	−0.004 (0.005)	0.27 (0.11)	**0.014**
AFLD-HF	0.293 (0.087)	**8.00E-04**	−0.001 (0.005)	0.574 (0.192)	**0.003**
AFLD-Hypertension	0.111 (0.05)	**0.027**	−0.004 (0.005)	0.216 (0.11)	**0.050**
NAFLD-AF	0.102 (0.07)	0.148	0.005 (0.005)	0.17 (0.14)	0.223
NAFLD-CAD	0.439 (0.12)	**2.00E-04**	0.001 (0.005)	0.784 (0.254)	**0.002**
NAFLD-HF	0.412 (0.149)	**0.006**	0.005 (0.005)	0.766 (0.277)	**0.006**
NAFLD-Hypertension	0.176 (0.066)	**0.008**	0.004 (0.006)	0.314 (0.134)	**0.019**
PBC-AF	−0.012 (0.042)	0.770	0.016 (0.008)	0.02 (0.049)	0.685
PBC-CAD	0.044 (0.043)	0.314	0.008 (0.008)	0.121 (0.376)	0.635
PBC-HF	0.09 (0.06)	0.132	0.013 (0.008)	0.2 (0.434)	0.470
PBC-Hypertension	0.046 (0.032)	0.145	0.009 (0.009)	0.101 (0.182)	0.450
PSC-AF	0.116 (0.043)	**0.007**	−0.003 (0.006)		
PSC-CAD	−0.052 (0.046)	0.261	0.001 (0.006)		
PSC-HF	0.001 (0.078)	0.988	−0.006 (0.006)		
PSC-Hypertension	−0.011 (0.043)	0.803	0.003 (0.008)		
VH-AF	−0.036 (0.061)	0.560	0.008 (0.006)	−0.091 (0.122)	0.456
VH-CAD	0.157 (0.063)	**0.014**	0.004 (0.005)	0.29 (0.18)	0.108
VH-HF	0.124 (0.09)	0.169	0.008 (0.005)	0.357 (0.225)	0.113
VH-Hypertension	0.084 (0.053)	0.112	−0.004 (0.006)	0.116 (0.12)	0.332

The genetic correlation was estimated using the LDSC method, while the genetic overlap was assessed using the HDL method. To account for multiple comparisons, a Bonferroni-corrected significance threshold was set at (p < 0.05/20 = 0.0025). Notably, we observed significant genetic correlations between AFLD and CAD, AFLD and HF, as well as NAFLD and CAD. These findings highlight the potential shared genetic components between these liver diseases and heart diseases.

### Shared gene loci for liver and heart disease

**Figure 1:**
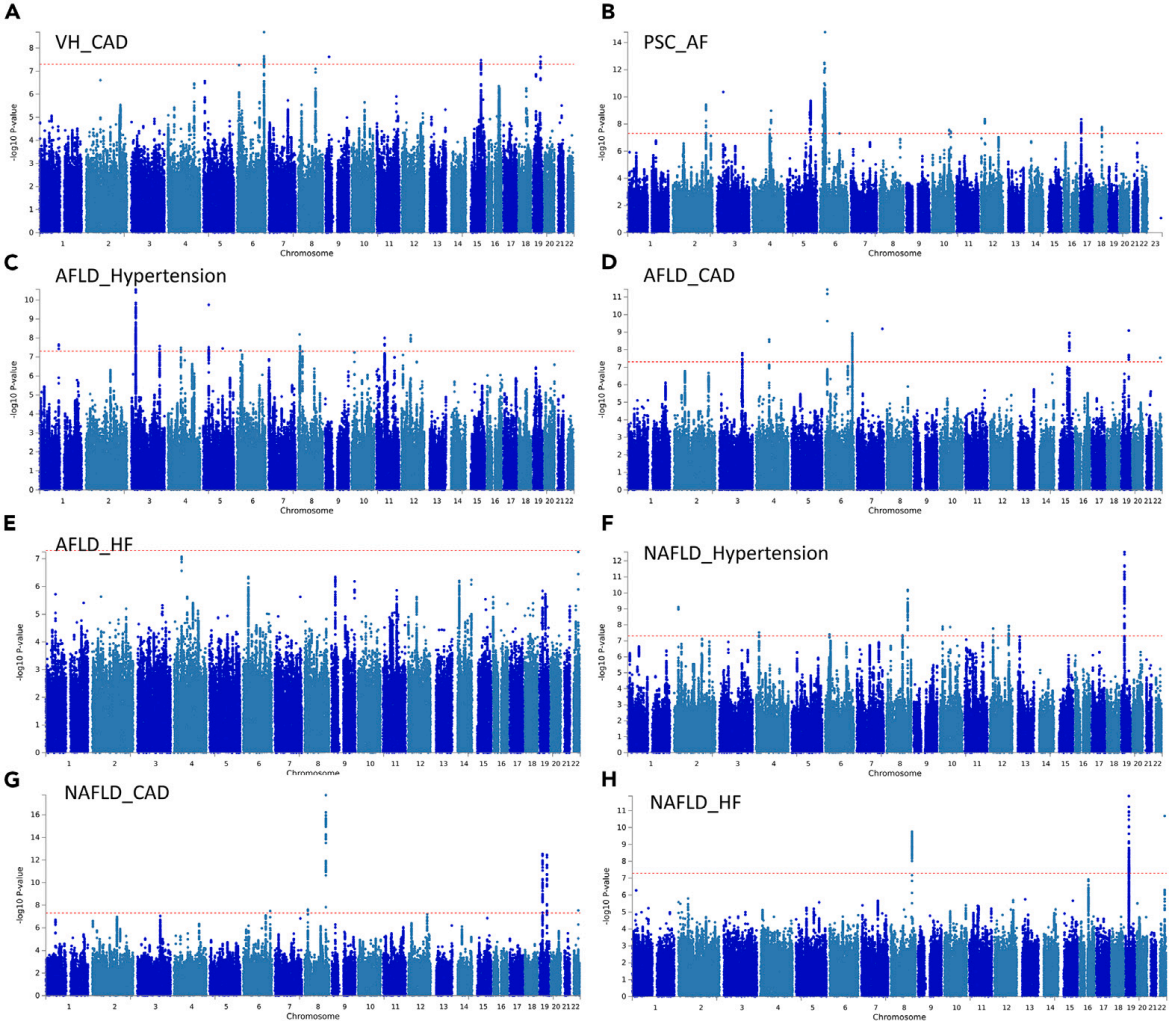
Manhattan plot depicting loci multiplicity between liver and heart diseases (A) VH-CAD. (B) PBC-AF. (C) AFLD-Hypertension. (D) AFLD-CAD. (E) AFLD-HF. (F) NAFLD-Hypertension. (G) NAFLD-CAD. (H) NAFLD-HF the Manhattan map for each disease pair displays the risk loci for pleiotropy, which are horizontally plotted on each chromosome from left to right. The -log10 transformed p value on the y axis indicates significance, with larger values indicating stronger statistical significance. The red line represents a threshold of 5E-8 for determining significant associations.

**Figure 2:**
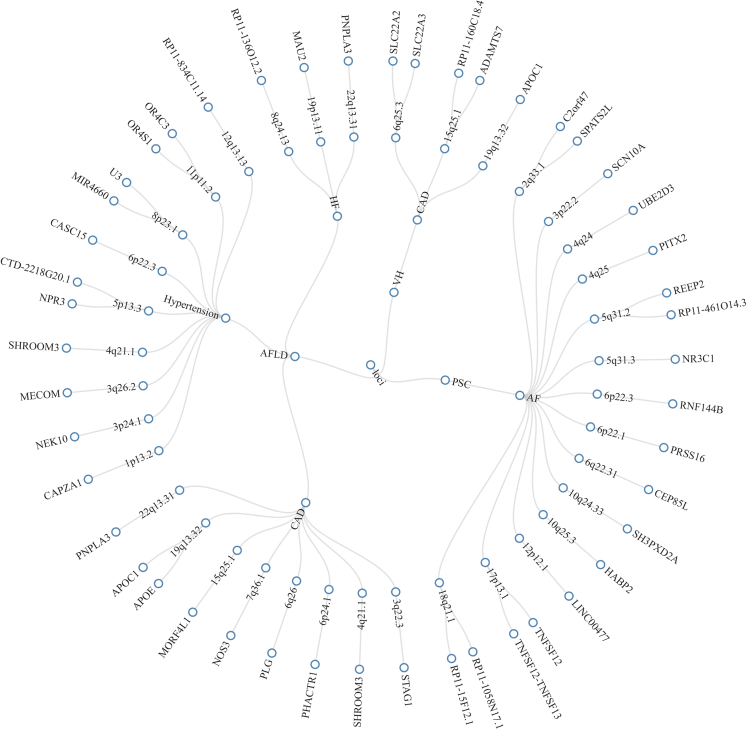
Characterizing the distribution of motifs in liver diseases and various cardiovascular disorders Figure 2 displays the shared loci among the AFLD-Hypertension, AFLD-HF, AFLD-CAD, VH-CAD, and PSC-AF disease groups. The outermost circle represents the gene names, while the second circle from the outside depicts the gene locus. For example, AFLD-CAD-19q13.32-APOC1, APOE indicates that 19q13.32 is a shared gene locus for this particular pair of diseases involving APOC1 and APOE.

Pleiotropy analysis (PLACO) was performed on 8 pairs showing genetic correlations, and the corresponding Manhattan plot is presented in Figure 1. Utilizing FUMA (functional mapping and annotation of GWAS), a total of 53 pleiotropic loci were identified and are depicted in Figure 2 (Table S2). Notably, several gene symbols such as PNPLA3 on Locus 22q13.31 (associated with AFLD-CAD, NAFLD-CAD, NAFLD-HF), RP11-136O12.2 on Locus 8q24.13 (associated with NAFLD-CAD, NAFLD-Hypertension, NAFLD-HF), APOE and APOC1 on Locus 19q13.32 (associated with AFLD-CAD, NAFLD-CAD), and SHROOM3 on Locus 4q21.1 (associated with AFLD-CAD, AFLD-Hypertension) were found to be associated with multiple pairs.

Then, we conducted quantile-quantile (Q-Q) mapping of pairs that were found to share a genetic locus, and the Q-Q plot did not indicate any genomic inflation (Figure S2). Additionally, Figure S3 presents essential information about each genomic risk locus, revealing a higher level of information richness in PSC-AF and NAFLD-Hypertension. The functional effects of pleiotropic SNPs on genes are depicted in Figure S4. Furthermore, through Bayesian co-localization analyses, we identified risk loci with causal effects on both diseases in Table 2. During this process, a total of 12 shared causal loci **(**PP.H4 > 0.7**)** were discovered for the two disorders. The significant expression of SHROOM3 on locus 4q21.1 in relation to AFLD-Hypertension (P = 3.26E-08, PP.H4 = 0.956) and AFLD-CAD **(**P = 2.65E-09, PP.H4 = 0.968) should be noted.

**Table 2:** Analysis of pleiotropic motifs and co-localization between liver disease and different cardiovascular diseases (p < 5E-8 & PPH4 > 0.70)

Trait pairs	Locus regions	p	Gene symbol	PP.H4
PSC-AF	4:103394218-104378012	2.57E-08	UBE2D3	0.854
PSC-AF	10:115329093-115516181	3.68E-08	HABP2	0.839
PSC-AF	17:7259087-7632621	4.41E-09	TNFSF12, TNFSF12-TNFSF13	0.82
AFLD-CAD	4:77019353-77494564	2.65E-09	SHROOM3	0.968
AFLD-Hypertension	4:77019353-77494564	3.26E-08	SHROOM3	0.956
NAFLD-CAD	8:126435663-126533955	1.83E-18	RP11-136O12.2	0.983
NAFLD-CAD	19:19260760-19865077	2.87E-13	TM6SF2	0.766
NAFLD-CAD	19:45332635-45428234	3.61E-13	APOE, APOC1	0.854
NAFLD-Hypertension	2:24696136-25502552	7.51E-10	DNAJC27	0.852
NAFLD-Hypertension	4:17552969-18177055	3.00E-08	LCORL	0.946
NAFLD-Hypertension	8:95679445-96112477	4.61E-08	NDUFAF6	0.867
NAFLD-Hypertension	19:19260760-19989679	2.79E-13	PBX4	0.745

In Table 2, a more comprehensive co-localization analysis was conducted for the disease pairs PSC-AF, AFLD-CAD, AFLD-Hypertension, NAFLD-CAD, and NAFLD-Hypertension to identify risk loci with causal effects on both diseases. SHROOM3 emerged as a shared genetic marker between AFLD-CAD, AFLD-Hypertension, and Hypertension in both disease groups.

### Results of MAGMA and HyPrColoc analyses

**Figure 3:**
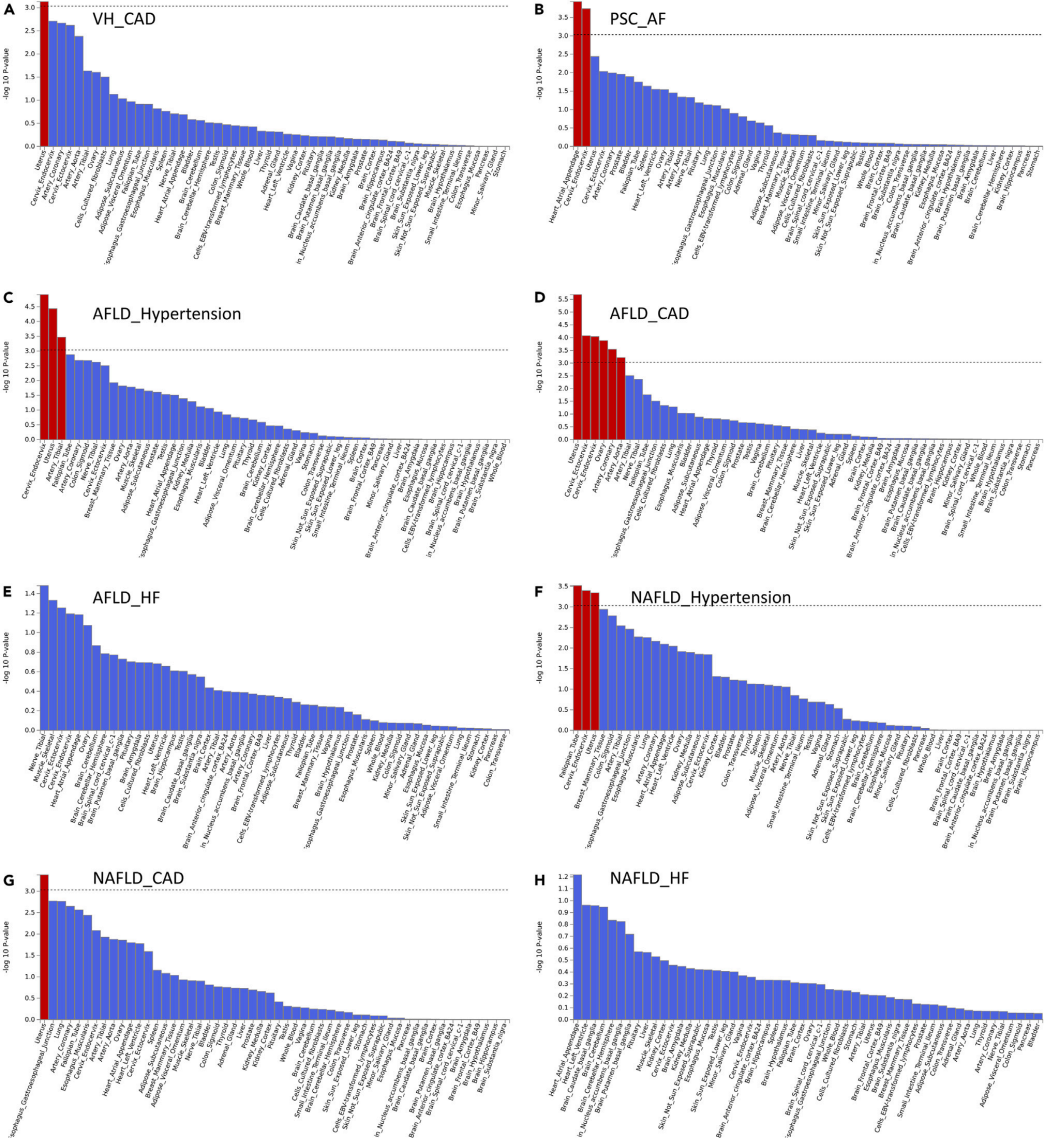
MAGMA analysis revealing enrichment of genetic overlap shared between disease pairs in GTEx v8 across 54 tissues According to the GTEx analysis, the following observations were made regarding the enrichment of different disease pairs in specific tissues. (A) VH-CAD analysis revealed an enrichment of this group of disease pairs in uterus tissue. (B) PSC-AF analysis indicated that this group of disease pairs exhibited enrichment in two tissues: heart-atrial-appendage and cervix-endocervix. (C) AFLD-Hypertension expression was found to be enriched in three tissues: cervix-endocervix, uterus, and artery-tibial. (D) AFLD-CAD demonstrated enrichment in several tissues, including uterus, cervix-ectocervix, cervix-endocervix, ovary, as well as artery-coronary and artery-aorta. (E) AFLD-HF did not demonstrate any significant enrichment in the relevant tissues. (F) NAFLD-Hypertension displayed enrichment in three tissues: fallopian tube, cervix-endocervix, and uterus. (G) NAFLD-CAD exhibited enrichment in three tissues: fallopian tube, cervix-endocervix, and uterus. (H) NAFLD-HF did not demonstrate any significant enrichment in the relevant tissues.

The tissue-specific MAGMA analysis revealed an intriguing observation that can be derived from Figure 3: PSC-AF shows high expression in cardiac tissues, while the majority of other disease pairs exhibit differential expression in the female reproductive system.

**Figure 4:**
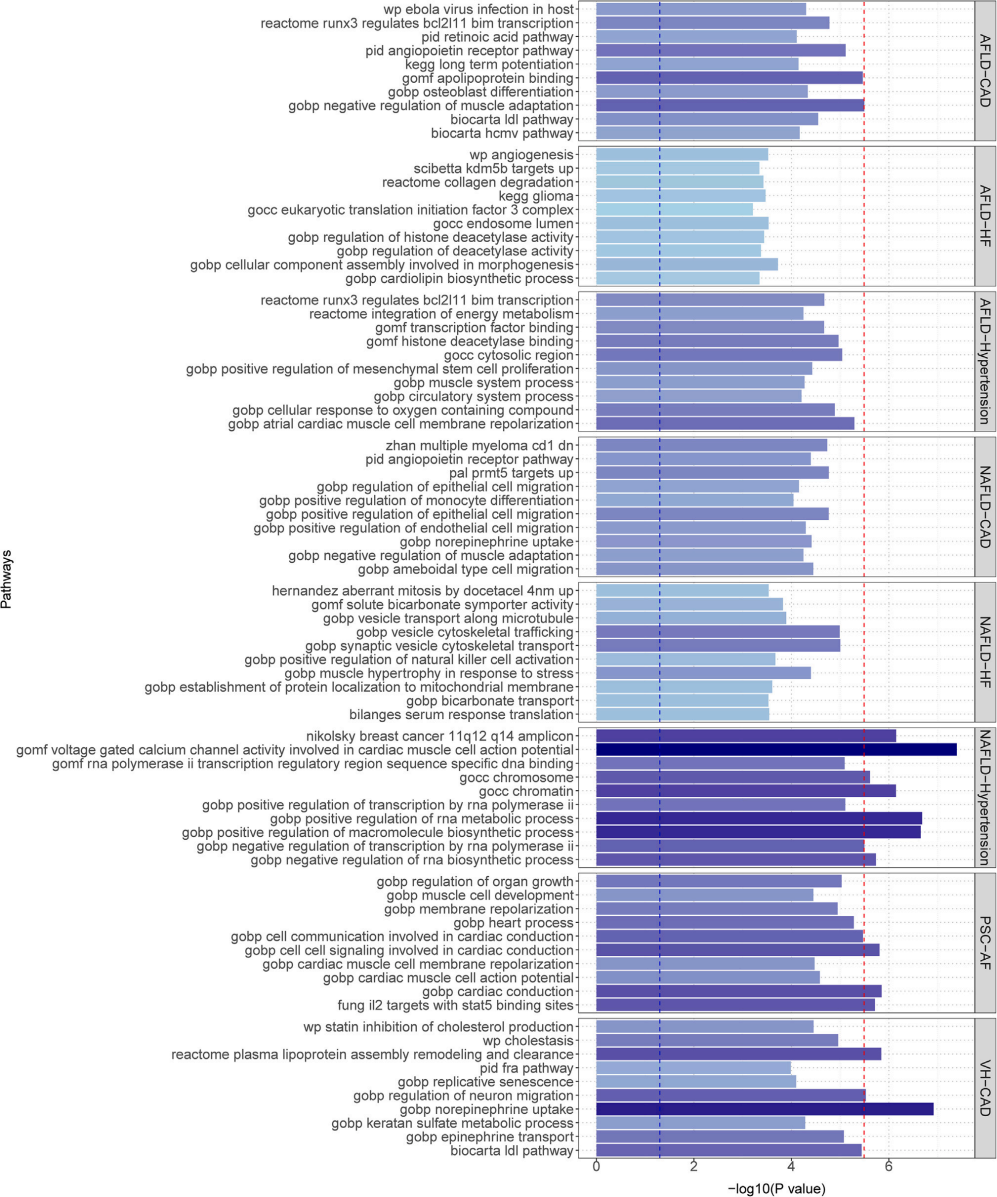
MAGMA analysis showing enrichment of shared genetic overlap between disease pairs in gene sets The P threshold at 0.0025 is represented by the red line. In NAFLD-Hypertension, several gene sets showed significant expression, including cardiac muscle cell action potential of gomf voltage-gated calcium channel activity, gobp positive regulation of RNA metabolic process, gobp positive regulation of macromolecule biosynthetic process, nikolsky breast cancer 11q12 q14 amplicon, and gocc chromatin. Notably, in VH-CAD, significant expression was observed in the gene sets of gobp norepinephrine uptake and reactome plasma lipoprotein assembly remodeling and clearance. Similarly, the gene set of gobp cardiac conduction exhibited significant expression in PSC-AF.

MAGMA gene set enrichment analysis was conducted on the multiplicity results, revealing the top 8 significantly enriched gene sets (Figure 4; Table S3). The majority of the significantly enriched gene set was found to be associated with the disease pair NAFLD-Hypertension, while the remaining genes were linked to the disease pairs PSC-AF and VH-CAD. Notably, this partial MAGMA gene set and tissue-specific analysis utilized the complete distribution of SNP p values.

Multi-trait co-localization HyPrColoc analysis identified 8 shared risk loci associated with liver and cardiovascular disease in immune cells (Table S4). These immune cells were localized in the following conditions: PSC-AF (Treg T cell: CD45RA^−^ CD28^−^ CD8br, GCST90001659), NAFLD-Hypertension (T cell: CD45RA^−^ CD4^+^, GCST90001534, TBNK cell: CD4^+^, GCST90001590, Monocyte: HLA DR on CD14^−^ CD16^+^, GCST90001984), AFLD-Hypertension (B cell: CD20^−^ CD38^−^, GCST90001423, B cell: CD20^−^ CD38^−^, GCST90001444), and AFLD-CAD (B cell: IgD+ CD24^+^, GCST90001412). Furthermore, sumheritable linkage disequilibrium score regression (S-LDSC) revealed heritability enrichment across multiple immune cell types (Figure S5; Table S5).

With the positional information of the lead SNPs, we identified genes associated with significantly linked pairs of pleiotropic risk loci (Tables S2 and S6). Some of these pleiotropic genes exhibited significant differential expression across multiple brain tissues (Figure S6; Table S7). The expression of HABP2, PLG, PNPLA3, and TM6SF2 was exclusively observed in hepatic tissues. Tissue-specific enrichment analysis revealed that these positionally mapped genes did not exhibit significant enrichment in any specific tissues even after applying multiple corrections (Figure S7). However, at a significance level of 0.05, pleiotropic genes were found to be significantly enriched in various brain tissues (Figure S8) for pathway enrichment analysis (Figure S9). The negative regulation of blood coagulation pathway (GO: 0030195) and DESCARTES LIVER HEPATOBLASTS exhibited high expression levels in the enrichment analysis.

The MAGMA gene test was conducted on the PLACO results. The Manhattan plot is depicted in Figure S10, while the Q-Q plot is illustrated in Figure S11. The Q-Q graph did not exhibit any evidence of gene inflation. By employing MAGMA gene analysis, we successfully identified 343 pleiotropic genes (p < 0.05/18645 = 2.682e-6), as presented in Tables S9 and S10. Furthermore, our tissue-specific enrichment analysis revealed significant enrichment of these location-mapped genes in the liver, cardiovascular, and pancreatic tissues (Figure S12). Pathway enrichment analysis can be observed in Figure S13, whereas cell type enrichment analysis is displayed in Figure S14. The nucleosome pathway (GO: 000786) exhibited significant enrichment in expression, along with TRAVAGLINI LUNG TERM2 DENDRTIC CELL and DURANTE ADULT OLFACTORY NEUROPITHELIUM MACROPHAGES.

### MR study

**Figure 5:**
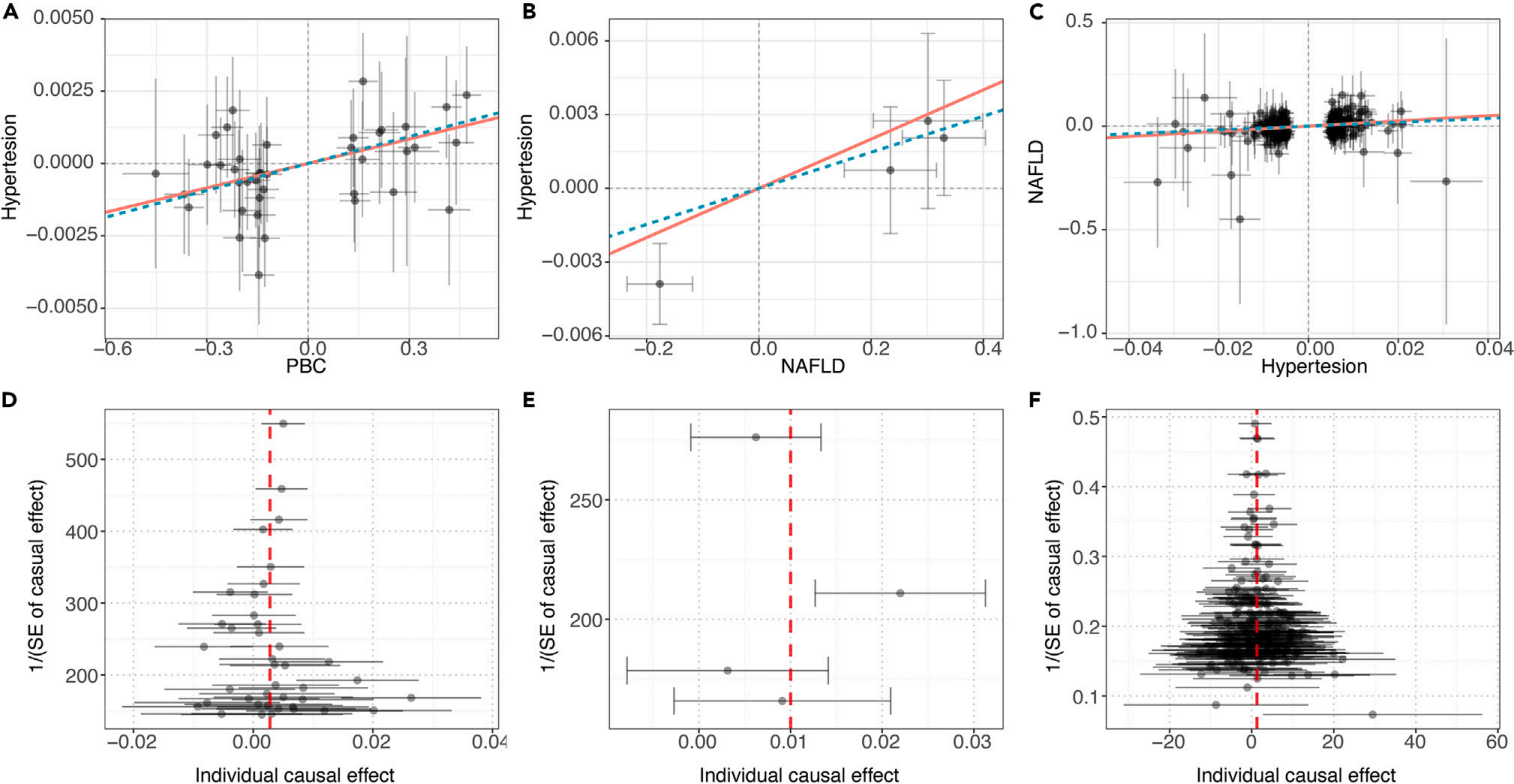
MR-PRESSO estimator and global test (A) The Mendelian randomized scatterplot of PBC-Hypertension illustrates PBC as the exposure factor and Hypertension as the outcome, showing no influence from outliers in Part A. (B) The Mendelian randomized scatterplot of NAFLD-Hypertension demonstrates NAFLD as the exposure factor and Hypertension as the outcome, with outliers having an impact. (C) The Mendelian randomized scatterplot of Hypertension-NAFLD in Part C indicates Hypertension as the exposure factor and NAFLD as the outcome, without any influence from outliers. (D) The Mendelian randomized funnel plot of PBC-Hypertension displays no heterogeneity between the two groups. (E) The Mendelian randomized funnel plot of NAFLD-Hypertension reveals significant heterogeneity between them. (F) The Mendelian randomized funnel plot for Hypertension-NAFLD demonstrates no heterogeneity between the two groups.

The causal relationship between the two types of diseases was inferred using the two-sample MR method. The summaries of instrumental variables and sensitivity analysis results are shown in Table S12. Genetically predicted Hypertension was causally associated with higher risks of NAFLD with an odds ratio (OR) of 3.482 (95% confidence interval [CI] = 1.889–6.420; p < 0.001). Genetically predicted PBC was causally associated with higher risks of Hypertension with an OR of 1.003 (95% CI = 1.001–1.005; p = 0.001). Genetically predicted NAFLD was causally associated with higher risks of Hypertension with an OR of 1.010 (95% CI = 1.002–1.018, p = 0.018). Various sensitivity analyses consistently supported these findings. Moreover, instrumental variables for AFLD were not identified at a significance level of p < 5E-8; therefore, the threshold was adjusted to 5E-6. Additionally, the intercept term in MR-Egger analysis showed no evidence of horizontal pleiotropy in the causal inference. Detailed results from all sensitivity analyses are presented in Table S13. The Hypertension-NAFLD, PBC-Hypertension, and NAFLD-Hypertension groups underwent additional MR-PRESSO analysis. The purpose of Figure 5 is to provide a more intuitive illustration of this concept. It was observed that the diseases, namely, PBC-Hypertension and Hypertension-NAFLD, exhibited robustness (Table S14).

## Discussion

In this genome-wide pleiotropic association study, we have identified a comprehensive and significant genetic association between liver and heart disease throughout the entire genome. Moreover, our thorough analyses have unveiled pleiotropic loci, potential shared biological pathogenic pathways, and underlying genetic factors associated with immunity, thereby establishing causal relationships in LHA. These findings collectively support the pivotal role of LHA in elucidating the common genetic etiology underlying both conditions.

Research has confirmed the undeniable correlation between liver and cardiovascular disease, which not only poses a significant burden on global healthcare and medical resources but also serves as a crucial factor in reducing quality of life and shortening life expectancy.^22^^,^^23^ The prevalence of liver disease results in an annual mortality rate of 2 million, accounting for 4% of all global deaths (equivalent to 1 in every 25 individuals).^24^^,^^25^ It is worth noting that males account for approximately two-thirds of fatalities associated with liver conditions. Globally, cardiovascular disease remains the most prevalent cause of mortality, contributing to an annual loss of over 60 million potential years of life in Europe.^26^^,^^27^ NAFLD has significantly contributed to the mortality and morbidity associated with cardiovascular diseases over the past two decades. The largest meta-analysis, encompassing observational, prospective, and retrospective studies involving 34,043 adults, revealed an OR of 1.64 (95% CI: 1.26–2.13) for fatal and/or non-fatal cardiovascular events in patients with NAFLD. And the risk escalates as the disease progresses.^22^^,^^28^^,^^29^ Therefore, the identification of shared genetic structures between liver and heart diseases is imperative to offer clinicians with innovative therapeutic insights.

The signal "communication" in LHA may result from immune stimulation and inflammatory responses, which are further attributed to genetic predispositions.^4^ According to the existing literature, it has been observed that Hypertension, CAD, and AF involve interconnected immune mechanisms in their occurrence and development. Moreover, they may also implicate more intricate signaling pathways. If these diseases continue to progress unabatedly, they will inevitably advance toward the advanced stage of cardiovascular disease known as HF. Similarly, liver disease exhibits a comparable pattern. Henceforth, we are eager to investigate whether there exists a shared genetic framework between cardiovascular disease and other prevalent or rare liver disorders.

In this study, the statistical significance of AFLD-CAD, AFLD-HF, and NAFLD-CAD in genetic association analysis remained robust even after applying the Bonferroni correction (p = 0.05/20), which is consistent with current understanding.^4^^,^^5^^,^^30^^,^^31^^,^^32^ Additionally, we have identified some intriguing disease pairs (p = 0.05), such as PSC-AF and VH-CAD, that are genetically linked. Regarding VH-CAD specifically, Wang et al. confirmed that patients with hepatitis exhibit an elevated risk of CAD, while Zhu et al. validated that viral infection with hepatitis A virus accelerates the onset of coronary atherosclerosis.^33^^,^^34^

The risk gene locus of shared causation highlights SHROOM3 as a notable candidate. While SHROOM3 has been implicated in various studies for its association with cardiovascular disease, its involvement in liver disease remains unreported. This discovery presents a promising genetic target for subsequent treatment strategies aimed at patients suffering from AFLD complicated by Hypertension and CAD.^35^^,^^36^

AF has been associated with immune modulation, and autoimmunity plays a crucial role in its development and persistence.^37^^,^^38^ PBC and PSC are linked to an increased incidence of cardiac dysfunction. This observation prompted the investigation of the connection between abnormal bile acid metabolism and cardiac pathology.^39^ Our study identified immune cells that target the functional relationship between AF and PSC. Although patients with PSC combined with AF have not yet been studied, our findings on the phenotype of Treg T cells provide new insights for future clinical diagnosis and treatment. Additionally, B cell therapy may be beneficial for patients with Hypertension combined with alcoholic liver. The case report conducted by Kazama et al. revealed that patients with AMA-M2-positive dilated cardiomyopathy had a higher likelihood of developing PBC and myocarditis, which were characterized by atrial dilation and arrhythmia (like AF).^40^ In these patients, steroid therapy demonstrated efficacy in suppressing myocardial inflammation and improving left ventricular ejection fraction.^41^ The findings of this study indicate the presence of a shared immune mechanism underlying both AF and PBC.

On tissue enrichment for expression, we found that several pairs were differentially expressed in the female genitalia. This may suggest to us that female patients may have a higher or lower risk of both liver and heart disease compared to male patients. This may need to be confirmed by subsequent large cohort studies.

The gene symbols like HABP2, PLG, PNPLA3, and TM6SF2 exhibit specific expression in hepatic tissue, and our study also suggests their potential impact on cardiovascular disease. The interaction between the aforementioned four gene symbols in LHA has not been verified through animal experiments. Instead, studies have been carried out at the population cohort level. Notably, Wu et al. have demonstrated a shared pathogenic role between NAFLD and CAD for variants of PNPLA3 and TM6SF2.^42^ Currently, the genetic mechanism underlying the association between NAFLD and coronary vascular disease (CVD) is well established and mature. PNPLA3 is predominantly expressed in hepatocytes and hepatic stellate cells, exhibiting hydrolytic activity against triglycerides (TGs) and retinol, thereby mitigating the risk of coronary heart disease.^22^^,^^43^ Franko et al. inferred a robust association between PNPLA3 variants and fatty liver, independent of insulin resistance, indicating that these two characteristics are distinct entities.^44^ It is reasonable to postulate that individuals with a deletion in the PNPLA3 gene may face an elevated susceptibility to cardiovascular disease. However, due to the presence of an I148M mutation in PNPLA3, it has been suggested that its association with an increased risk of cardiovascular events is not always consistent, and a definitive causal relationship between NAFLD and CVD cannot be established.^45^^,^^46^ The TM6SF2 gene, belonging to the transmembrane 6 superfamily member 2, serves as a determinant for hepatic TG content, serum aminotransferases, low-density lipoprotein cholesterol (LDL-C), and TG levels.^22^^,^^47^ The activity of TM6SF2 is essential for the normal secretion of very low density lipoprotein (VLDL), and impaired function of TM6SF2 significantly contributes to the development of NAFLD. The gene exerts its influence on the occurrence and progression of cardiovascular diseases by modulating the lipid metabolism pathway.^48^ Our study diverges from prior research; the gene symbols PLG and HABP2 were identified as the pivotal factor in regulating LHA in this study. The role of PLG and HABP2 in liver fibrosis enhancement is acknowledged, while its cardiovascular effects remain uncertain. Although variants of PLG have been demonstrated to exhibit a correlation with coronary events in individuals with CAD who are undergoing statin treatment, the precise underlying mechanism remains yet to be elucidated.^49^ In the future, HABP2 may emerge as a promising therapeutic target for patients with hepatocardiac diseases.

In the pathway analysis, we observed a remarkable enrichment of the nucleosome pathway, which aligns with the findings reported by Lo Re O et al., thereby reinforcing the evidence of pleiotropy in our study. The research team conducted a study involving 120 clinical subjects and discovered a significant association between elevated levels of circulating nucleosomes and both NAFLD and adverse cardiovascular events.^50^ Nucleosomes facilitate genome compaction and safeguarding within cellular nuclei, while their composition and post-translational modifications intricately govern gene expression. Individuals with NAFLD often exhibit dysregulated metabolism, leading to increased cellular apoptosis and subsequent elevation of nucleosomes. Thus, the involvement of nucleosome metabolic pathways serves as compelling evidence for pleiotropy in the pathogenesis of LHA. But the interpretation of the results pertaining to cell type (like TRAVAGLINI LUNG TERM2 DENDRTIC CELL and DURANTE ADULT OLFACTORY NEUROPITHELIUM MACROPHAGES) enrichment remains ambiguous. Further investigations are imperative to validate the findings. In a sense, it indirectly lays the groundwork for potential therapeutic and intervention strategies targeting LHA.

In the MR study, as part of enhancing the robustness of the evidence of the LHA, we observed a reciprocal causal relationship between Hypertension and NAFLD. The aforementioned statement is in line with the MR findings of Yuan et al. and can serve as substantiation to support the presence of reciprocal causation involving LHA.^51^ The existence of PBC-Hypertension is associated with a positive causal relationship, potentially mediated by immune damage, necessitating further investigation through more diverse MR analysis.^52^

Some animal experiments have also demonstrated the existence of an intricate interaction mechanism between the liver and heart. Kato et al. substantiated the inseparable correlation between HF and liver metabolism using a rat model of cachexia.^53^ Our study further confirmed the presence of genetic associations in disease pairs such as AFLD-HF (p = 8.00E-04) and NAFLD-HF (p = 0.006).

Recently, prospective cohort studies are being conducted to investigate the underlying mechanism of hepatocardiac comorbidity. Isaak et al. investigated the association between cirrhosis and cardiac involvement using state-of-the-art liver and heart magnetic resonance imaging (MRI) techniques. In their well-designed prospective study, they included 42 cirrhotic participants of diverse origins and 18 control participants. A comprehensive MRI examination of both the heart and liver was conducted during a single imaging session. These findings strongly support the hypothesized pathophysiological mechanisms underlying cirrhotic cardiomyopathy; the inflammation and fibrosis of the liver can induce the release of vasodilators and inflammatory mediators, which subsequently leads to a reduction in central blood volume accompanied by high dynamic circulation. These hemodynamic and inflammatory alterations contribute to the development of cirrhotic cardiomyopathy.^54^

### Limitations of the study

The study is subject to inherent limitations. Given the restricted access to individual-level data and the lack of a substantial cohort, our investigations heavily depend on publicly available GWAS databases for analysis. As a result, it is impractical to conduct additional subgroup or stratified analyses (incorporating lifestyle or environmental factors) in order to evaluate potential influences on liver and heart disease. The primary emphasis of this study was placed upon common genetic variants, which might inadvertently neglect rare or low-frequency variants that could exhibit noteworthy associations with liver and cardiovascular diseases. Additionally, variations in the quality control process and covariate inclusion exist among different datasets. Our investigation is confined solely to samples originating from individuals of European descent; hence its applicability across various racial populations remains uncertain. The identification of novel pathway signals and gene loci in our study necessitates further validation through clinical cohort studies or related animal experiments to enhance the robustness of our findings. The exclusion of certain liver and heart diseases from our study due to sample size limitations might have resulted in missed opportunities for discovering potentially significant associations.

### Conclusions

The summary of our findings based on GWAS data reveals a shared genetic architecture between liver and heart diseases, highlighting the underlying biological mechanisms involving immunity and signaling pathways. Furthermore, employing polytropic loci analysis strengthens these associations. Importantly, utilizing MR methodology confirms the presence of a causal relationship among disease pairs. These results provide novel insights for clinical diagnosis and treatment strategies, enabling targeted interventions in patients with LHA.

## STAR★Methods

### Key resources table

**Table:** 

REAGENT or RESOURCE	SOURCE	IDENTIFIER
**Deposited data**		

Non-alcoholic fatty liver disease (NAFLD)	Finngen R9	https://r9.finngen.fi/
Viral hepatitis (VH)	Finngen R9	https://r9.finngen.fi/
alcoholic liver disease (AFLD)	FinnGen R9	https://r9.finngen.fi/
Primary biliary cholangitis (PBC)	Cordell HJ et al.^55^	https://ega-archive.org/
Primary sclerosing cholangitis (PSC)	Cordell HJ et al.^55^	https://ega-archive.org/
Heart failure (HF)	GWAS	https://www.ebi.ac.uk/gwas/
Coronary artery disease (CAD)	GWAS	https://www.ebi.ac.uk/gwas/
Hypertension	GWAS	https://www.ebi.ac.uk/gwas/
Atrial Fibrillation (AF)	GWAS	https://www.ebi.ac.uk/gwas/
Immunocell	ImmGen	https://www.immgen.org/

**Software and algorithms**		

PLACO	Ray, D. et al.^56^	https://github.com/RayDebashree/PLACO
MAGMA	de Leeuw, C.A. et al.^57^	https://ctg.cncr.nl/software/magma
FUMA	Watanabe, K. et al.^58^	https://fuma.ctglab.nl/
Coloc amd Hyprcoloc	Hormozdiari, F. et al.^59^ and Foley, C.N. et al.^60^	https://www.rdocumentation.org/packages/coloc/versions/5.1.0
PLINK	Purcell, S. et al.^61^	https://www.cog-genomics.org/plink/
MendelianRandomization	Burgess, S. et al.^62^	https://github.com/topics/mendelianrandomization
R	R Foundation for Statistical Computing	https://www.r-project.org/

### Resource availability

#### Lead contact

Further information and requests for resources should be directed to and will be fulfilled by the lead contact, Shudong Xia (shystone@zju.edu.cn).

#### Materials availability

This study did not generate new unique reagents, paper or reproduce the results is available from the lead contact upon request.

#### Data and code availability

• This paper analyzes existing, publicly available data. These access URLs for the datasets are listed in the key resources table.
• This paper does not report original code.
• Any additional information required to reanalyze the data reported in this paper is available from the lead contact upon request.

### Experimental model and study participant details

Our study is computational that does not use experimental models typical in the life sciences.

### Method details

#### Study population and data collection

##### Summary statistics of liver diseases

Genotype data from 8,021 cases and 16,489 controls across five European cohorts were incorporated by PBC, and the resulting estimates were subjected to fixed-effects meta-analysis using the META package.^55^ The dataset for primary sclerosing cholangitis (PSC) was derived from one of the largest genome-wide association studies (GWAS) on PSC, which included a total of 4,796 cases and 19,955 population controls and identified four new loci with genome-wide significance.^63^

NAFLD comprised 2,275 cases and 37,552 controls; VH comprised 2,143 cases and 375,134 controls; AFLD comprised 2,761 cases and 366,450 controls.^64^ FinnGen collected genomic and health data from a cohort of half a million Finnish Biobank participants in order to provide novel medical insights as well as establish world-class resources for future research endeavors. The model included gender, age, ten genetic principal components, and genotyping batch as covariates (Refer to Table S1).

##### Summary statistics on cardiovascular diseases

The results of the GWAS meta-analysis for HF included a total of 47,309 cases and 930,014 controls. Using the fixed-effects IVW method in METAL, we performed a meta-analysis of the summarized data which identified 12 independent SNPs associated with HF.^65^ The CAD data originated from the GWAS conducted on 34,541 CAD cases and 261,984 controls sourced from the UK Biobanking Resource. This was further validated by replication in an additional cohort consisting of 88,192 cases and 162,544 controls from CARDIoGRAMplusC4D. Ultimately, our meta-analysis identified a total of 75 loci that reached genome-wide significance.^66^ The data pertaining to Hypertension were obtained through a study investigating common genetic associations among age-related diseases involving a total of 129,909 cases and 354,689 controls.^67^ The AF data was derived from a large-scale GWAS encompassing over one million individuals including both AF cases (60,620) and controls (970,216) (Refer to Table S1 for data details).^68^

##### Immunocell summary data

Access to a comprehensive collection of 292 immune cell types from the ImmGen consortium is available, encompassing B cells, gamma delta T cells, alpha beta T cells, innate lymphocytes, myeloid cells, stromal cells, and stem cells.^69^ Additionally, GWAS summary statistics for 731 additional immunophenotypes can be accessed through the GWAS catalog (IDs from GCST0001391 to GCST0002121) (Complex genetic signatures in immune cells underlie autoimmunity and inform therapy). The immunological features include absolute cell counts (n = 118), median fluorescence intensity reflecting surface antigen levels (n = 389), morphologic parameters (n = 32), and relative cell counts (n = 192). These features encompass B cells, CDCs, T-cell maturation stage, monocytes, myeloid cells. The morphologic parameter feature includes the CDC and TBNK panels. The GWAS data for immune traits were derived from a cohort of 3,757 Europeans with covariates such as sex and age considered. Approximately 22 million SNPs were genotyped using high-density arrays across a reference panel based on Sardinian sequences.

##### Linked disequilibrium score regression

The genetic correlation analyses were performed using the standard analytical procedures for linked disequilibrium score regression (LDSC) and high-definition likelihood (HDL).^70^^,^^71^ These analyses aimed to assess the shared polygenic structure between traits, with LD scores calculated from European pedigree samples obtained from the Thousand Genomes Project serving as a reference panel.^72^ We have implemented stringent quality control measures for SNPs. The genetic correlation analyses assessed the shared polygenic structure between traits, where LD scores were calculated from European pedigree samples obtained from the Thousand Genomes Project, which served as a reference panel. For SNPs, we implemented strict quality control measures: (i) excluding non-bipartite allele SNPs and those with strand-ambiguous alleles; (ii) excluding SNPs without rs tags; (iii) removing duplicates or SNPs that were not included in the 1,000 Genomes Project or had mismatched alleles; and (iv) due to their complex LD structure, excluding SNPs located within the major histocompatibility complex region (chr6: 28.5-33.5Mb) from the LDSC analysis. Additionally, only SNPs with a minor allele frequency (MAF) > 0.01 were retained. We did not impose any restrictions on the intercept in the LDSC analysis, which not only accounts for residual confounding but also indicates whether there was potential sample overlap.

##### The genetic enrichment analysis of immune cells

We conducted further investigation into the enrichment of SNP heritability in liver and heart diseases using various immune cell data through stratified LD score regression to evaluate the possibility of significant genetic enrichment of specific cell types in these tissues. After adjusting for the baseline model and all gene sets, we assigned a P-value to the regression coefficient *Z* score to determine the significance of SNP heritability enrichment estimates in each tissue and cell.

##### PLACO analysis

The SNP-Level PLACO approach is a novel method for investigating pleiotropic loci between complex traits based solely on summarized genotype-phenotype association statistics.^56^ We computed the squared Z scores for each variant and excluded SNPs with excessively high Z^2^ values (>80). Furthermore, considering the potential correlation between liver and heart disease, we estimated the correlation matrix of Z. Additionally, we calculated the correlation matrix of the Z scores for each variant. Subsequently, we assessed the hypothesis of no pleiotropy using the Intersection-Union Test (IUT). The final p-value of the IUT test was determined as the maximum value between H0 and H1.

##### MAGMA analysis

Based on the PLACO results, we conducted a comprehensive mapping of the identified loci to nearby genes in order to investigate the shared biological mechanisms underlying these pleiotropic loci. We performed Generalized Gene-Set Analysis of GWAS Data (MAGMA) analysis on genes located within or overlapping with pleiotropic loci, using both PLACO output and single-trait GWAS data, to identify potential candidate pleiotropic genes. The significance threshold for MAGMA analysis was set at P < 0.05/N_genes_ = 3E -06.^57^

To determine the biological functions of these multi-functional motifs, we employed Functional Mapping and Annotation of Genome-Wide Association Studies (FUMA).^58^ Additionally, pathway enrichment analyses were conducted based on the Molecular Signature Database (MSigDB) to elucidate the functional roles of mapped genes.^73^ It is worth noting that SNPs and risk motifs located in MHC regions were excluded from both PLACO and MAGMA gene/gene-set analyses/.

A Bayesian co-localization analysis was conducted using the R package coloc to identify potential shared causal variants corresponding to paired traits for each pleiotropic locus that were annotated by FUMA.^59^ The co-localization analysis is based on the assumption of a single causal variant, and it will provide posterior probabilities (PP) for the five hypotheses associated with each polytomous locus For FUMA-annotated pleiotropic loci, we conducted Bayesian co-localization analyses using the R package coloc to further identify potential shared causal variants for each pleiotropic locus corresponding to paired traits.

##### Hypothesis prioritization for multi-trait colocalization

The HyPrColoc (hypothesis prioritization for multi-trait colocalization) analysis, in conjunction with immunohistochemistry GWAS, was employed to underscore the pivotal role of immune cells in the progression of liver and heart diseases.^60^

##### Mendelian randomization

We independently screened all significant loci associated with exposure as instrumental variables (P<5×10^-8^) using the clump program in PLINK software, with a r^2^ threshold of 0.001 and a window size of 10,000 kb.^61^ To ensure the robustness of our instrumental variables, we computed both the coefficient of determination r^2^ and F statistic for each one. The F-statistic was calculated as follows: where r^2^ represents the proportion of variance explained by the instrumental variable, n denotes the sample size, and k indicates the number of SNPs.

### Qualification and statistical analysis

#### Statistics and software

For all diseases, we obtained the European-only summary statistics and conducted rigorous quality control measures: (i) excluded non-bipartite allelic SNPs and those with ambiguous alleles on the strand; (ii) excluded SNPs without rs tags; (iii) removed duplicated SNPs or those not included in the 1000 Genomes Project, or with mismatched alleles; (iv) excluded SNPs located within the region of the major histocompatibility complex (chr6: 28.5-33.5Mb) due to their intricate LD structure; and (v) retained SNPs with a minor allele frequency (MAF) > 0.01. Due to different consortia providing data for liver disease and cardiovascular disease, there was minimal sample overlap between them. Finally, relevant information for each SNP such as effect size, standard error, effect allele, and P value was preserved for further analysis.

The co-localization analysis is based on the assumption of a single causal variant and provides posterior probabilities (PP) for five hypotheses: (1) H0: neither trait is genetically associated in the region; (2) H1: only trait 1 is genetically associated in the region; (3) H2: only trait 2 is genetically associated in the region; (4) H3: both traits are related but with different causal variants; and (5) H4: both traits are correlated and share a common causal variant. We utilized the coloc.abf function for co-localization analysis with significance thresholds set at p1 = p2 = 1 × 10^-4^ and p12 = 1 × 10^-5^.

The primary approach employed in Mendelian randomization is inverse variance weighting (IVW). The instrumental variable (IV) must satisfy three assumptions: (1) the IV should exhibit correlation with the exposure; (2) the IV should not be associated with confounding factors of the exposure and outcome associations; and (3) the effect of the IV on the outcome is solely mediated by the exposure.Additionally, we conducted several sensitivity analyses. Firstly, Q-tests using IVW and MR-Egger were employed to detect potential violations of the hypothesis by assessing heterogeneity among individual instrumental variables.^74^ Secondly, MR-Egger was used to estimate horizontal pleiotropy based on its intercept, ensuring that genetic variation was independently associated with both exposure and outcome.^62^ Furthermore, we enhanced the stability and reliability of our findings by conducting additional analyses (weighted median and weighted mode) under different modeling assumptions and strengths within the framework of Mendelian randomization.

The statistical analyses were conducted using R 4.3.1 software, while the MR analyses were performed utilizing the MendelianRandomization package.^75^ This study adhered to the Strengthening the Reporting of Genetic Association Studies (STREGA) reporting guideline.

## Supplemental information

Data S1. Tables S1–S14

Data S2. Source code
