# Supplementary material for: Shared genetic architecture and causal relationship between liver and heart disease
Source: iScience. 2024 Mar 6;27(4):109431. doi: 10.1016/j.isci.2024.109431 (PMC10959668; doi:10.1016/j.isci.2024.109431)
Supplement: Document S1. Figures S1–S14 [file mmc1.pdf]

iScience, Volume *27*

Supplemental information

Shared genetic architecture and causal

relationship between liver and heart disease

Ziyi Fang, Sixiang Jia, Xuanting Mou, Zhe Li, Tianli Hu, Yiting Tu, Jianqiang Zhao, Tianlong Zhang, Wenting Lin, Yile Lu, Chao Feng, and Shudong Xia

Supplement1

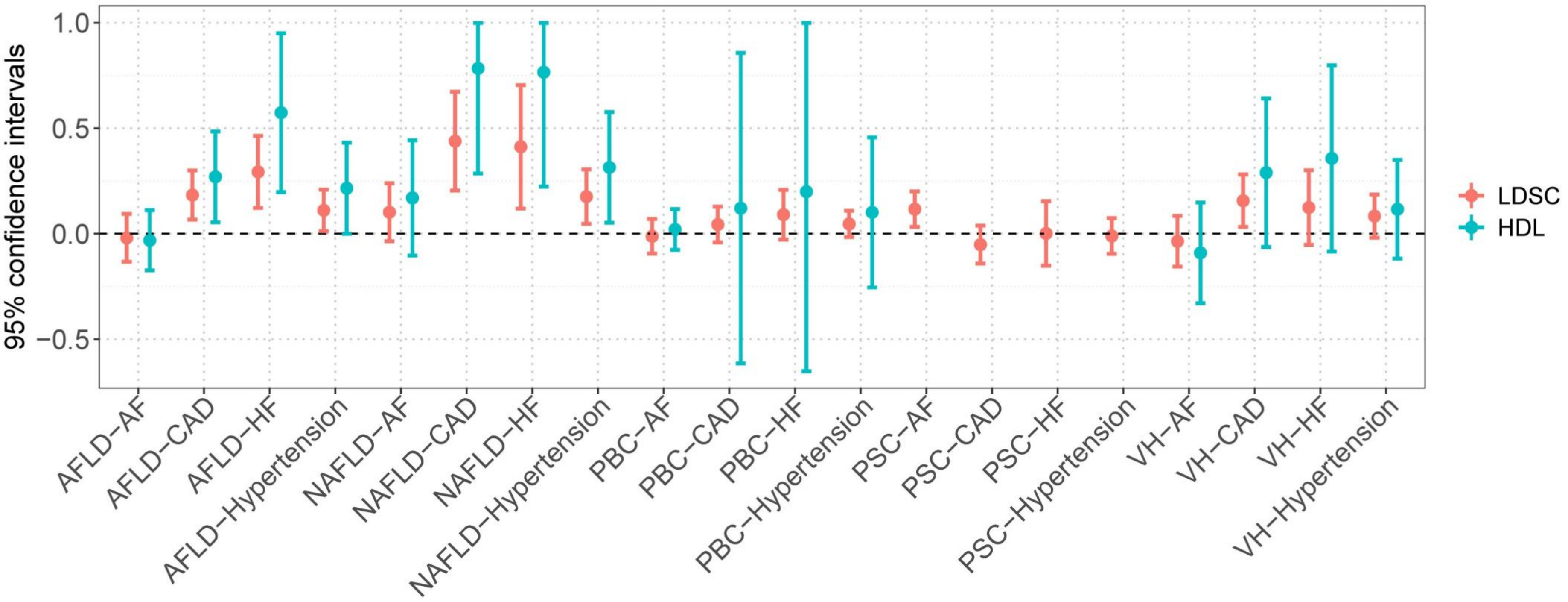

Figure S1. Genetic correlation analysis between liver and cardiovascular disease, related to Table 1

In Figure S1 we can intuitively find that there are significant genetic correlations between AFLD-CAD, AFLD-HF, AFLD-Hypertension, NAFLD-CAD , NAFLD-HF , NAFLD-Hypertension, PSC-AF and VH-CAD. (Note: The associations with upper and lower confidence intervals exceeding 1 or -1 were constrained to be set at 1 or -1)

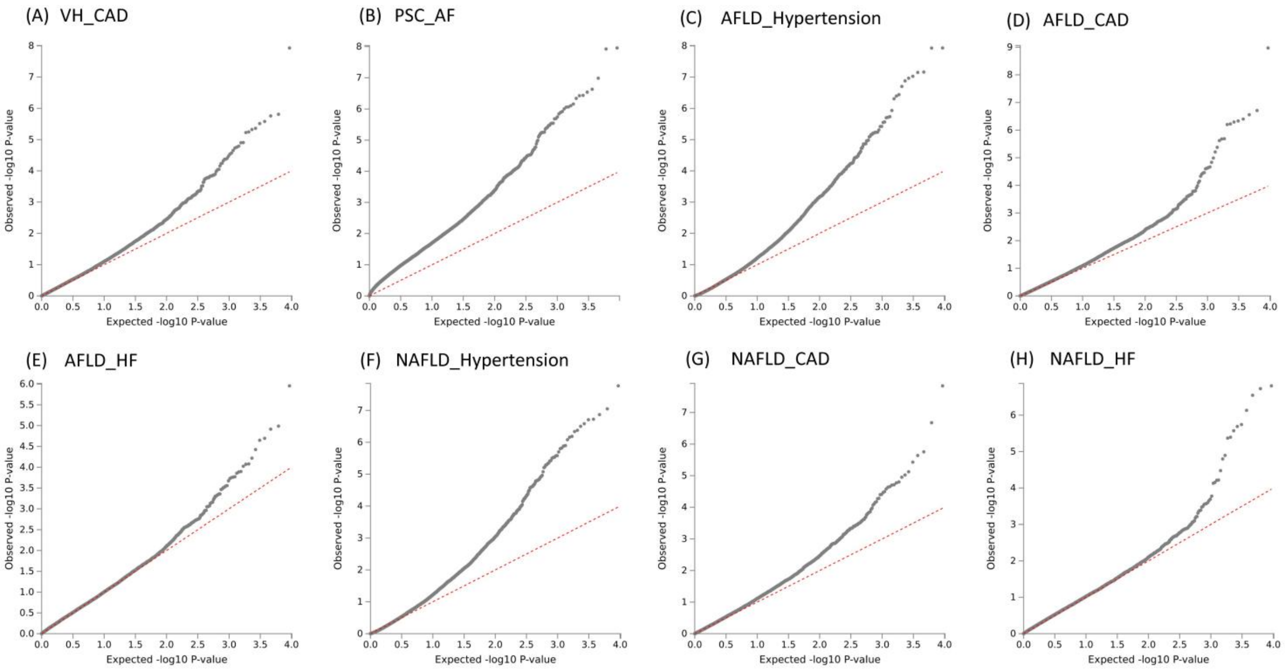

Figure S2. QQ plot for multiple validity analysis, related to Table 1

A.VH-CAD, B.PSC-AF, C.AFLD-Hypertension, D.AFLD-CAD, E.AFLD-HF, F. NAFLD-Hypertension, G.NAFLD-CAD, H.NAFLD-HF. The genetic association between LDSC and HDL was further investigated to explore potential pleiotropy, no gene expansion was observed in disease pairs.

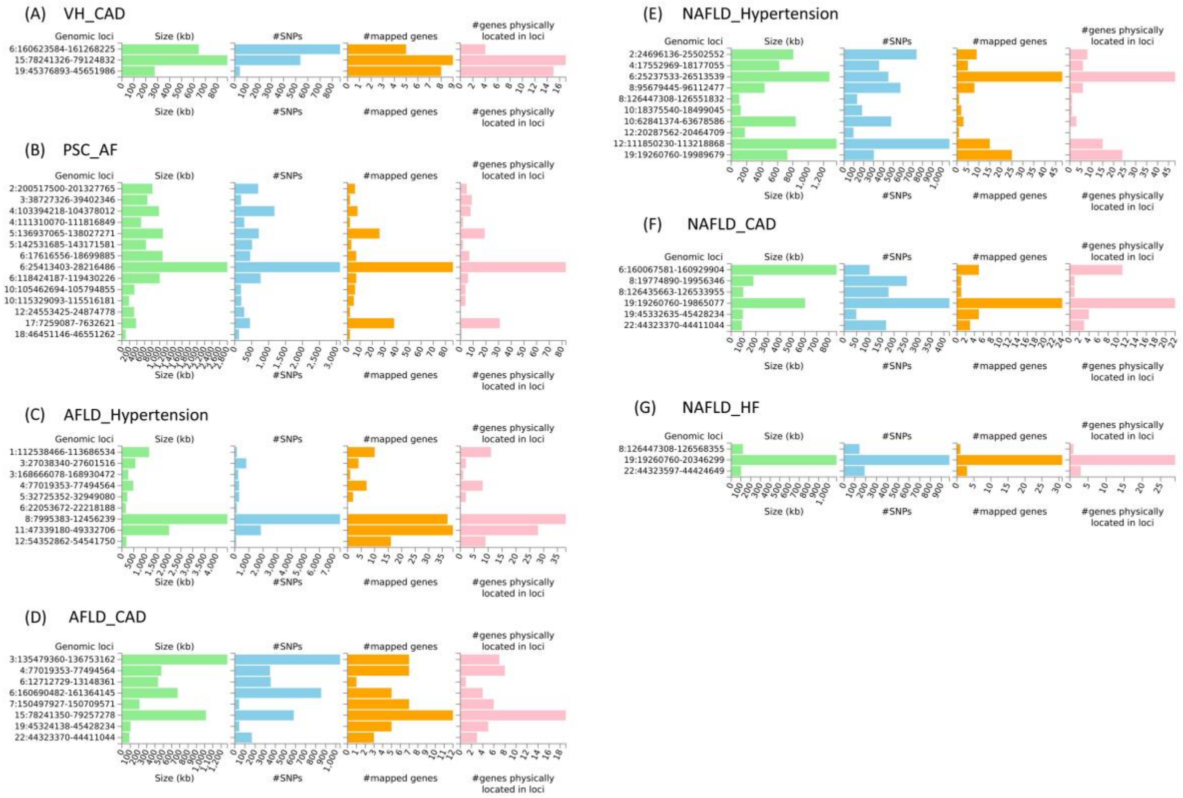

Figure S3. Specific information on polytropic loci, related to Table 2 and Table S2

A.VH-CAD, B.PSC-AF, C.AFLD-Hypertension, D.AFLD-CAD, E.AFLD-HF, F. NAFLD-Hypertension, G.NAFLD-CAD. The size of the risk locus, the number of SNPs, the number of Map genes, and the count of genes located within the locus are arranged in a left-to-right manner. The ordinate represents the specific loci where gene pleiotropy is observed in this cluster of diseases.

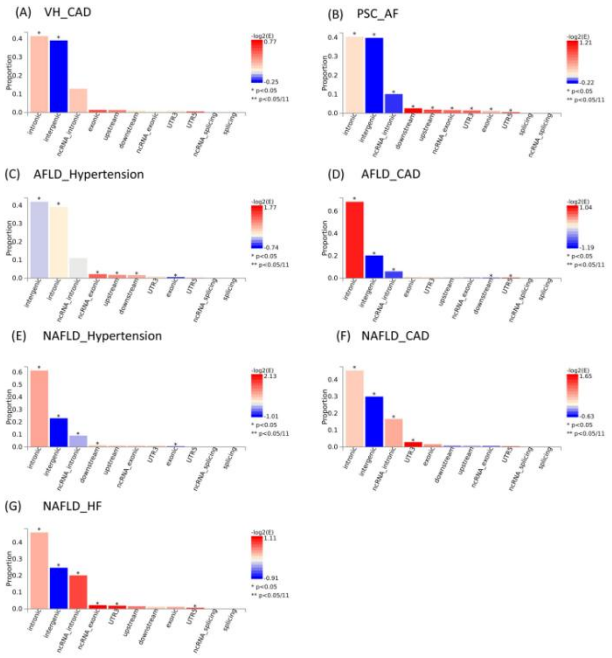

Figure S4. Functional effects of polyvalent SNPs on genes, related to Table 2 and Table S2

A.VH-CAD, B.PSC-AF, C.AFLD-Hypertension, D.AFLD-CAD, E.AFLD-HF, F. NAFLD-Hypertension, G.NAFLD-CAD. The horizontal coordinate in each subgroup of Figure S4 represents various genomic regions, including intronic, intergenic, NcRNA - intronic exonic, upstream and downstream, ncRNA - exonic, UTR3, UTR5, ncRNA_splicing, and splicing. In this representation scheme, the color red indicates positive feedback while blue indicates negative feedback. Additionally, asterisks (*) denote statistical significance at $P < 0.05$ level and double asterisks (**) indicate statistical significance at a Bonferroni-corrected threshold of $P < 0.05/11$.

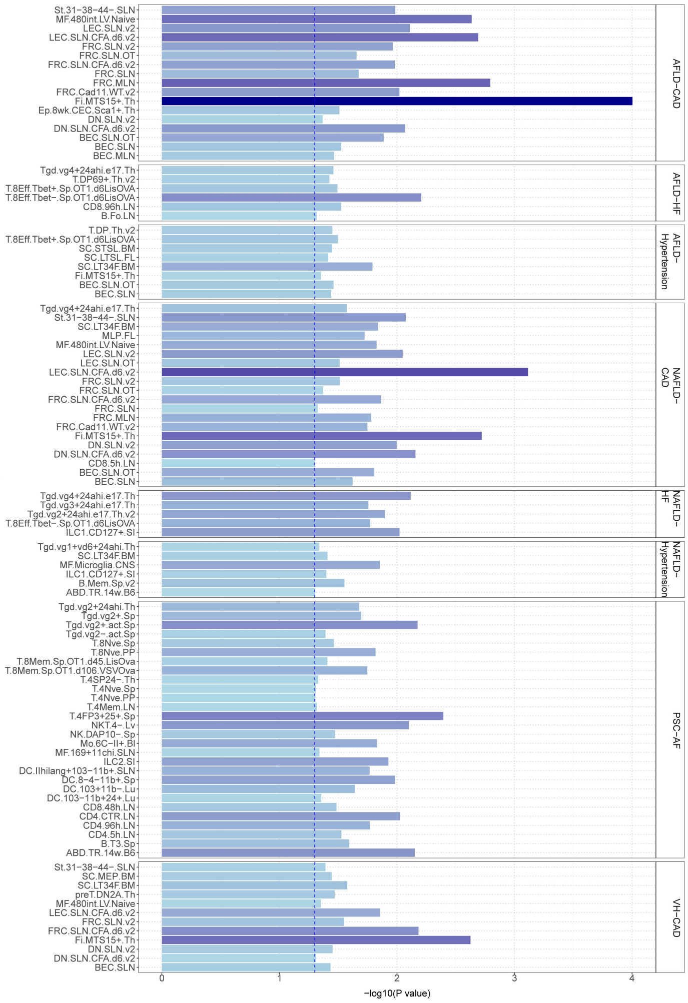

Figure S5. S-LDSC enrichment analysis identifies immune cells with significant heritability enrichment for liver and cardiovascular disease, related to Table 2 and Table S5

The blue line in Figure S5 represents a significance level of p=0.05, while the left side of the ordinate corresponds to the gene enrichment expression set for each disease pair. For example, the following gene sets exhibited high expression levels in AFLD-CAD, Fi.MTS15+.Th, FRC.MLN, LEC.SLN.CFA.d6.v2, MF.480int.LV.Naive, LEC.SLN.v2, dn.sln.cf.d6.v2, FRC.Cad11.WT.v2, St.31-38-44-.sln, frc.sln.cfa.d6.v2, frc.sln. v2, BEC. Sln. OT,FRC.SLN.frc.sln. OT,BEC.SLN,E p .8wk.CEC.Sca1+.Th,BEC.MLN,DN.SL N .v2.The expression of Fi.MTS15+.th was the most significant one among them.

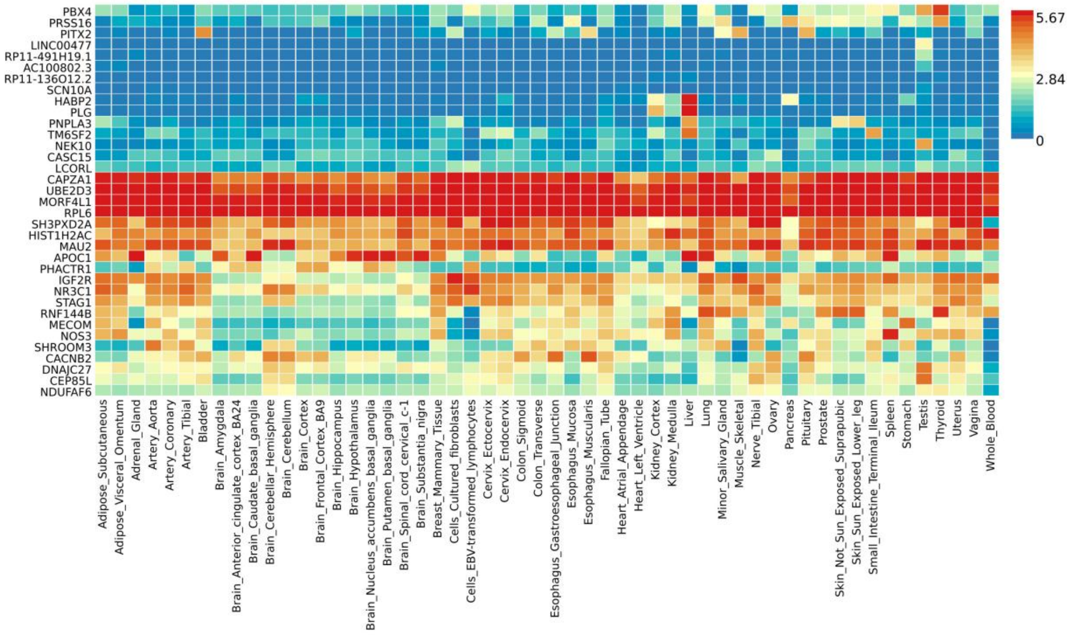

Figure S6. Expression of pleiotropic genes in 54 tissues of GTEx v8, related to Table 2, Table S2 and Table S6

The horizontal axis in Figure S6 represents the tissues, while the vertical axis represents the genotype. Red indicates a positive correlation, whereas blue indicates a negative correlation. Our findings reveal that HABP2, PLG, PNPLA3, and TM6SF2 exhibit high expression levels in liver tissue.

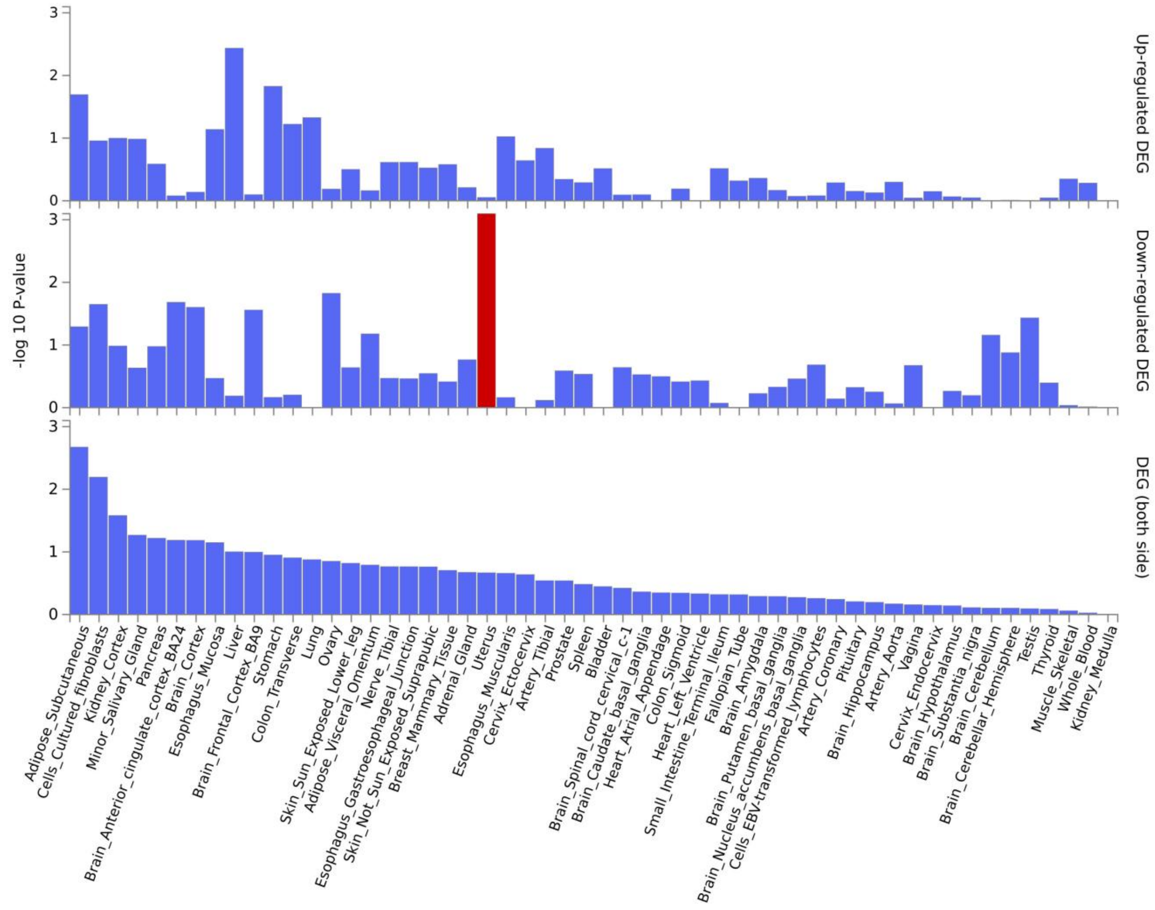

Figure S7. Results of enrichment analysis of gene sets for polytropic genes, related to Table 2 and Table S8

The horizontal axis in Figure S7 represents different tissues, while the right vertical axis represents gene expression analysis in upstream and downstream regions. It is evident that the most significant difference in expression analysis occurs in the Uterus.

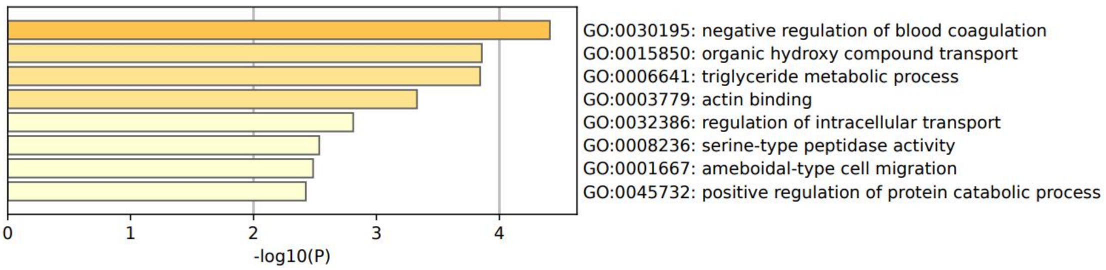

Figure S8. Pathway enrichment of pleiotropic genes (Positional Matching), related to Table 2 and Table S6

The enrichment analysis of pleiotropic pathway matched with gene location in Figure S8 revealed significant expression of the pathway GO: 0030195: Negative Regulation of Blood Coagulation.

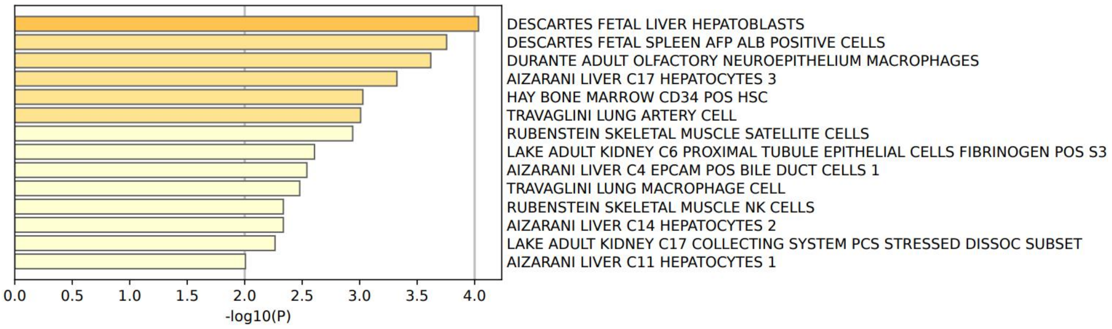

Figure S9. Cell-type enrichment of pleiotropic genes (Positional Matching), related to Table 2 and Table S6

The cell enrichment analysis in Figure S9 revealed a significant expression of DESCARTES FETAL LIVER HEPATOBLASTS within this specific cell type, as matched with gene location.

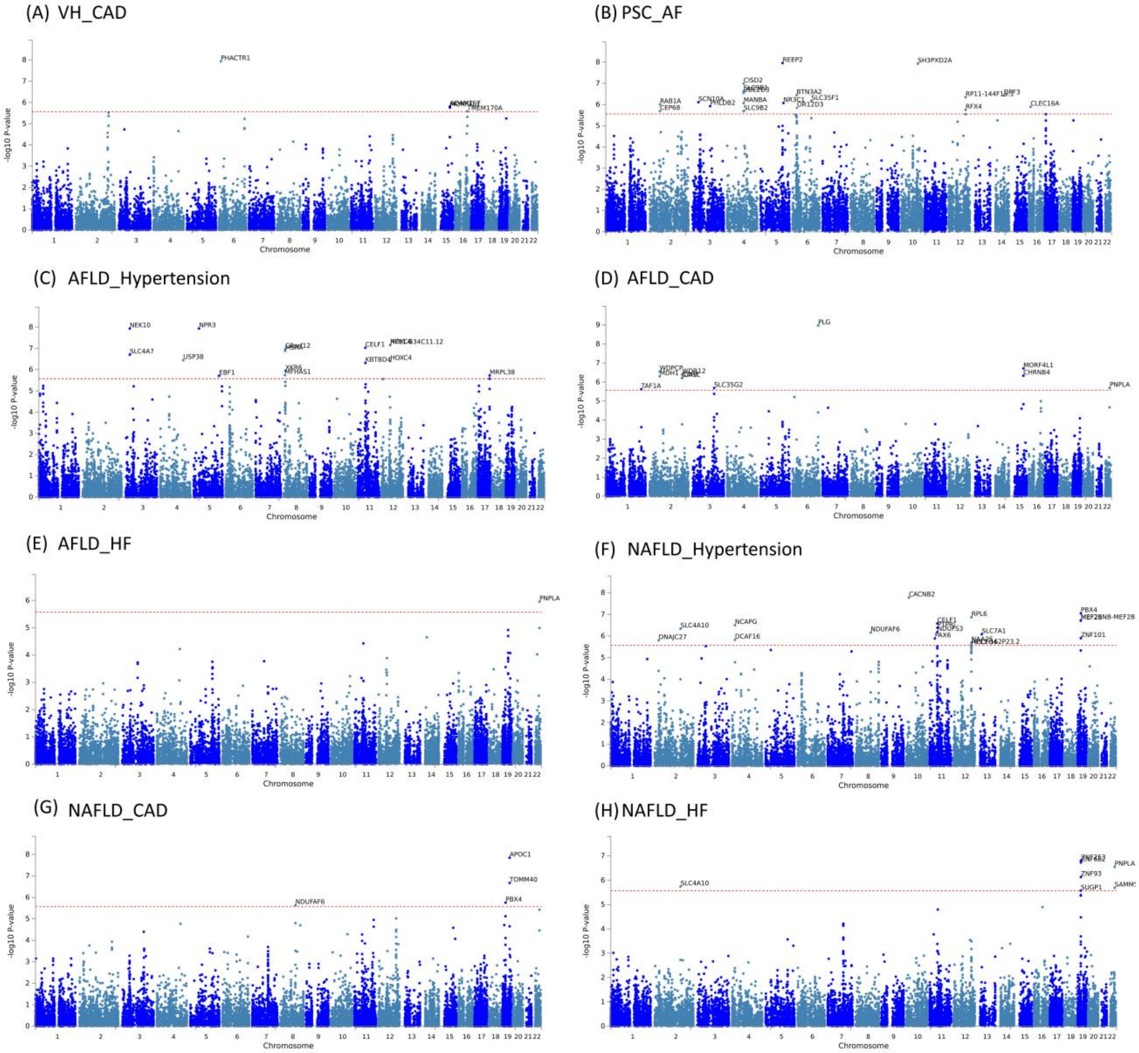

Figure S10. Manhattan plot of Magma gene analysis between disease pairs, related to Figure 3 and Table S9

A.VH-CAD, B.PSC-AF, C.AFLD-Hypertension, D.AFLD-CAD, E.AFLD-HF, F. NAFLD-Hypertension, G.NAFLD-CAD. H.NAFLD-HF. The Figure S10 illustrates an increasing number of significant pleiotropic sites for PSC-AF, AFLD-Hypertension, AFLD-CAD, and NAFLD-Hypertension. Note：Red line represents significance after multiple corrections

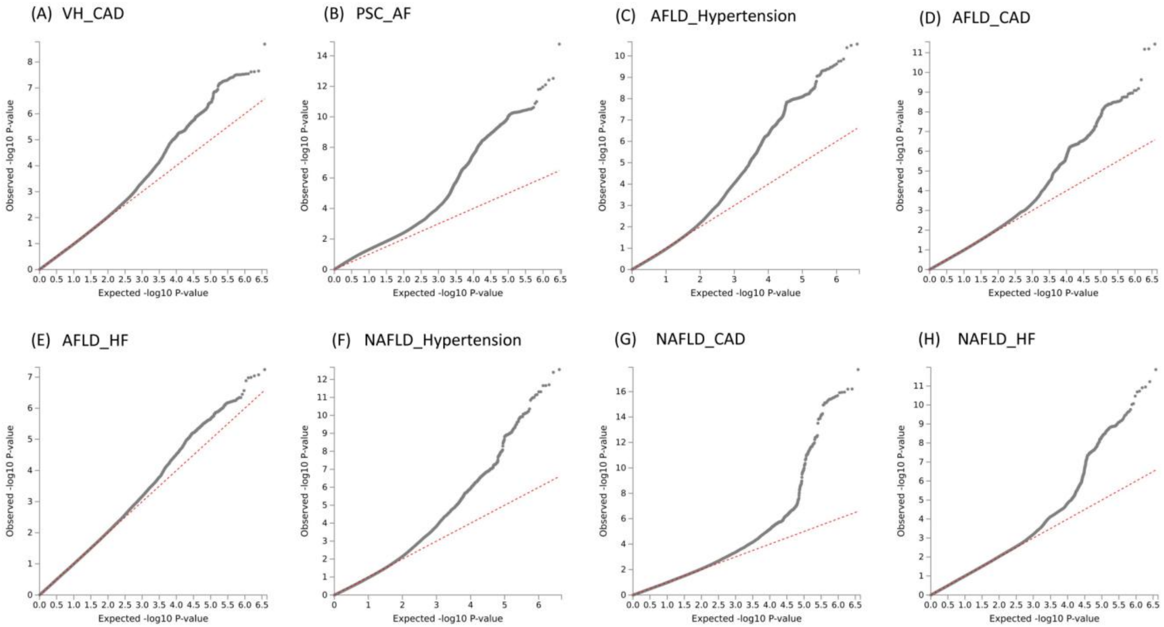

Figure S11. QQ plot of Magma gene analysis between disease pairs, related to Figure 3 and Table S9

A.VH-CAD, B.PSC-AF, C.AFLD-Hypertension, D.AFLD-CAD, E.AFLD-HF, F. NAFLD-Hypertension, G.NAFLD-CAD. H.NAFLD-HF. The QQ map in Figure S10 was utilized to depict the MAGMA gene enrichment analysis for various diseases, no gene expansion was observed in disease pairs.

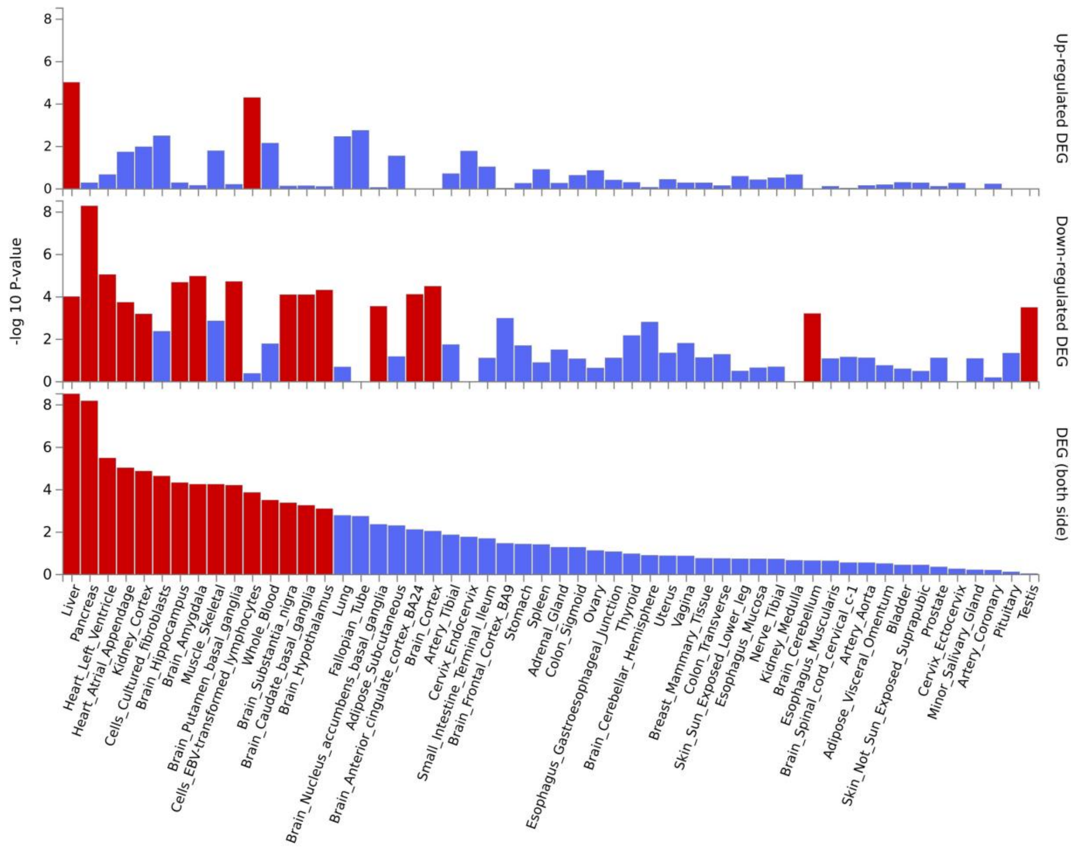

Figure S12. Enrichment of pleiotropic genes (based on Magma gene test) in different tissues,related to Figure 4 and Table S11

The gene pleiotropy enrichment analysis conducted by Magma in Figure S12 revealed that gene regulation was observed both upstream and downstream of liver tissue, while the remaining genes exhibited expression patterns in various other tissues such as pancreas, heart, kidney, brain, among others.

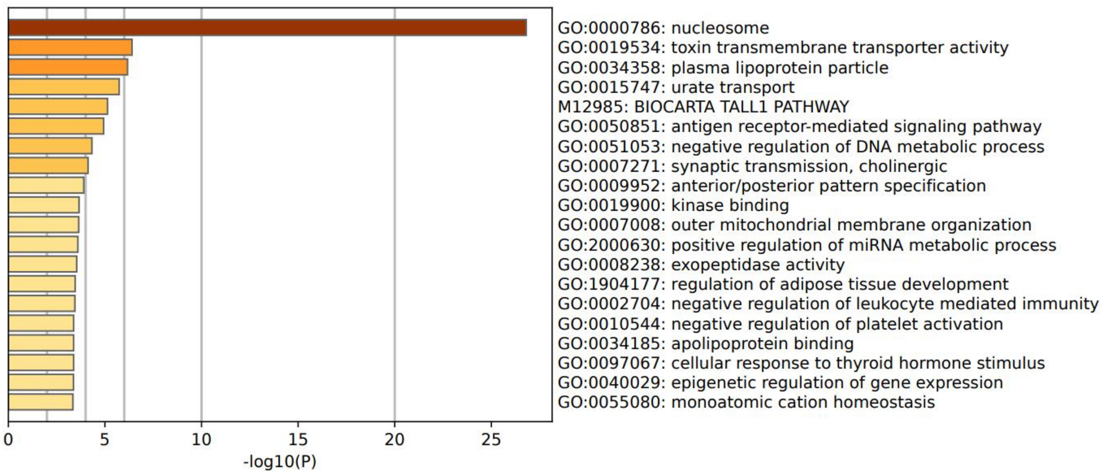

Figure S13. Pathway enrichment of pleiotropic genes (based on Magma gene test), related to Figure 4, Table S9 and Table S10

The analysis of genetic pleiotropy pathway based on Magama analysis revealed a significant enrichment of nucleosome (GO:0000786) in Figure S13.

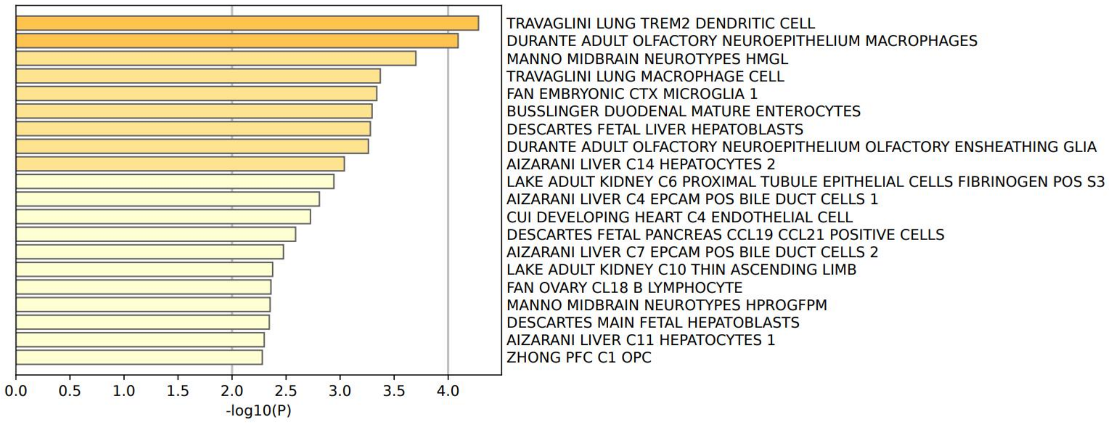

Figure S14. Cell-type enrichment of pleiotropic genes (based on Magma gene test)related to Figure 4， Table S9 and Table S10

The analysis of genetic pleiotropy, based on Magama analysis, revealed a significant enrichment of TRAVAGLINI LUNG TERM2 DENDRTIC CELL and DURANTE ADULT OLFACTORY NEUROPITHELIUM MACROPHAGES in Figure S14.
